# Endoscopic resection of a duodenal duplication cyst: A case report

**DOI:** 10.1002/deo2.88

**Published:** 2022-01-09

**Authors:** Sayumi Kurita, Kazuo Kitagawa, Naoki Toya, Masahiko Kawamura, Muneo Kawamura, Ken Eto

**Affiliations:** ^1^ Department of Surgery The Jikei University Kashiwa Hospital Chiba Japan; ^2^ Department of Surgery Kawamura Hospital Shizuoka Japan; ^3^ Department of Surgery The Jikei University School of Medicine Tokyo Japan

**Keywords:** duodenal duplication cyst, endoscopic resection, hot snare polypectomy

## Abstract

A duodenal duplication cyst (DDC) is a rare congenital anomaly. Gastrointestinal duplication cysts are traditionally treated by complete surgical resection due to the potential precancerous conditions. Here, we describe an asymptomatic DDC that was successfully treated using endoscopic resection. A submucosal tumor in the descending portion of the duodenum was detected in a 71‐year‐old female during a regular checkup at our hospital. Upper gastrointestinal endoscopy showed a 10‐mm pedunculated submucosal tumor. Endoscopic ultrasonography revealed a 10‐mm cystic tumor of low echogenicity that included nodules and debris. Endoscopic resection with hot snare polypectomy was performed for diagnosis and treatment. The postoperative course was uneventful. Histologic examination revealed that the cystic tumor was a DDC. Endoscopic resection is a safe, effective, and minimally invasive alternative to surgical resection for small DDCs with malignant potential.

## INTRODUCTION

A duodenal duplication cyst (DDC) is a rare congenital anomaly usually encountered during infancy or early childhood.^1^ Gastrointestinal duplication cysts (GIDCs) occur mainly in the distal ileum, followed by the esophagus, colon, and jejunum. DDCs account for only 2%–12% of all GIDCs.^2^ DDCs are only occasionally detected in adults and are usually asymptomatic. Common clinical features include duodenal obstruction, hemorrhage, biliary obstruction, cyst infection, and pancreatitis. Although DDCs are generally benign, malignant changes can occur rarely in adults. Asymptomatic cysts are usually managed expectantly but some authorities recommend intervention based on potential complications, including malignant transformation.^3^ The ideal treatment for DDC is complete surgical resection, but this procedure has a high rate of morbidity. Thus, the use of endoscopic resection is increasingly used to treat DDCs. Here, we report the case of a 71‐year‐old woman with an asymptomatic DDC who was successfully treated endoscopically.

## CASE REPORT

**FIGURE 1:**
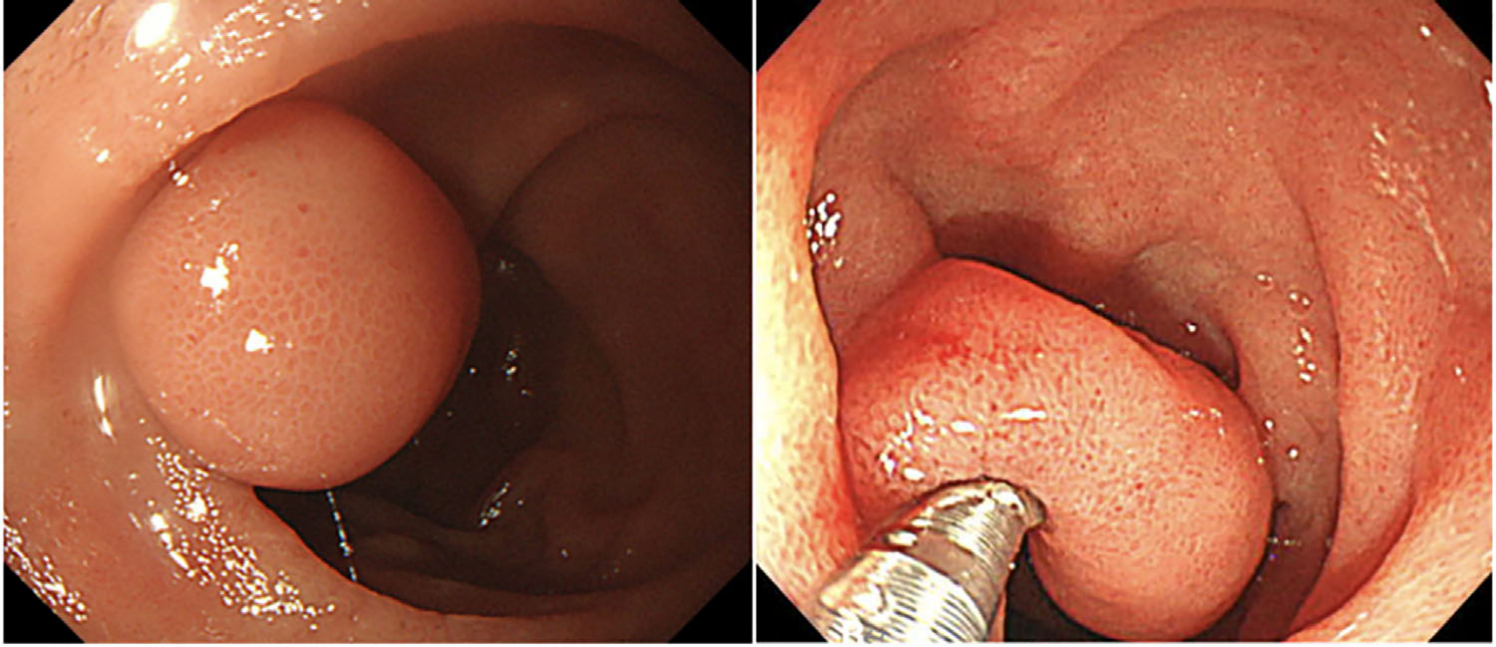
Endoscopic images of the duodenal duplication cyst. The cyst was soft and easily movable by bioptome. The 10‐mm pedunculated submucosal tumor was located in the descending portion of the duodenum, next to the papilla of Vater

A 71‐year‐old female visited our hospital for a regular checkup, whereupon a submucosal tumor (SMT) in the descending portion of the duodenum was detected. She had a medical history of hypertension and dyslipidemia. Her family and social history were unremarkable. Physical examination revealed no palpable mass, and all clinical laboratory data were normal. Upper gastrointestinal (GI) endoscopy showed a soft pedunculated 10‐mm SMT, which was easily movable by the bioptome. The SMT was located in the descending portion of the duodenum, next to the papilla of Vater (Figure 1).

**FIGURE 2:**
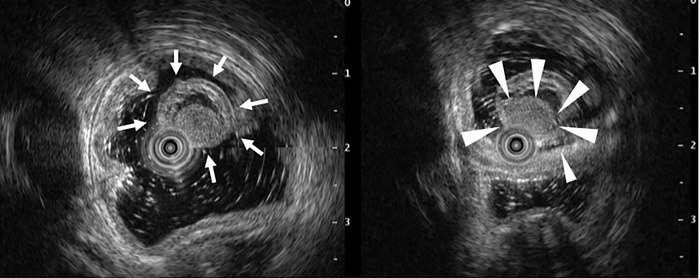
Endoscopic ultrasonography of the duodenal duplication cyst. A 10‐mm cystic tumor (white arrow) of low echogenicity that included an isoechoic circular structure (white arrowhead). The first layer of the cyst lumen was hyperechoic, the second layer was hypoechoic, the third layer was hyperechoic, the fourth layer was hypoechoic, and the fifth layer was hyperechoic. The circular structure was thought to be nodules and debris

**FIGURE 3:**
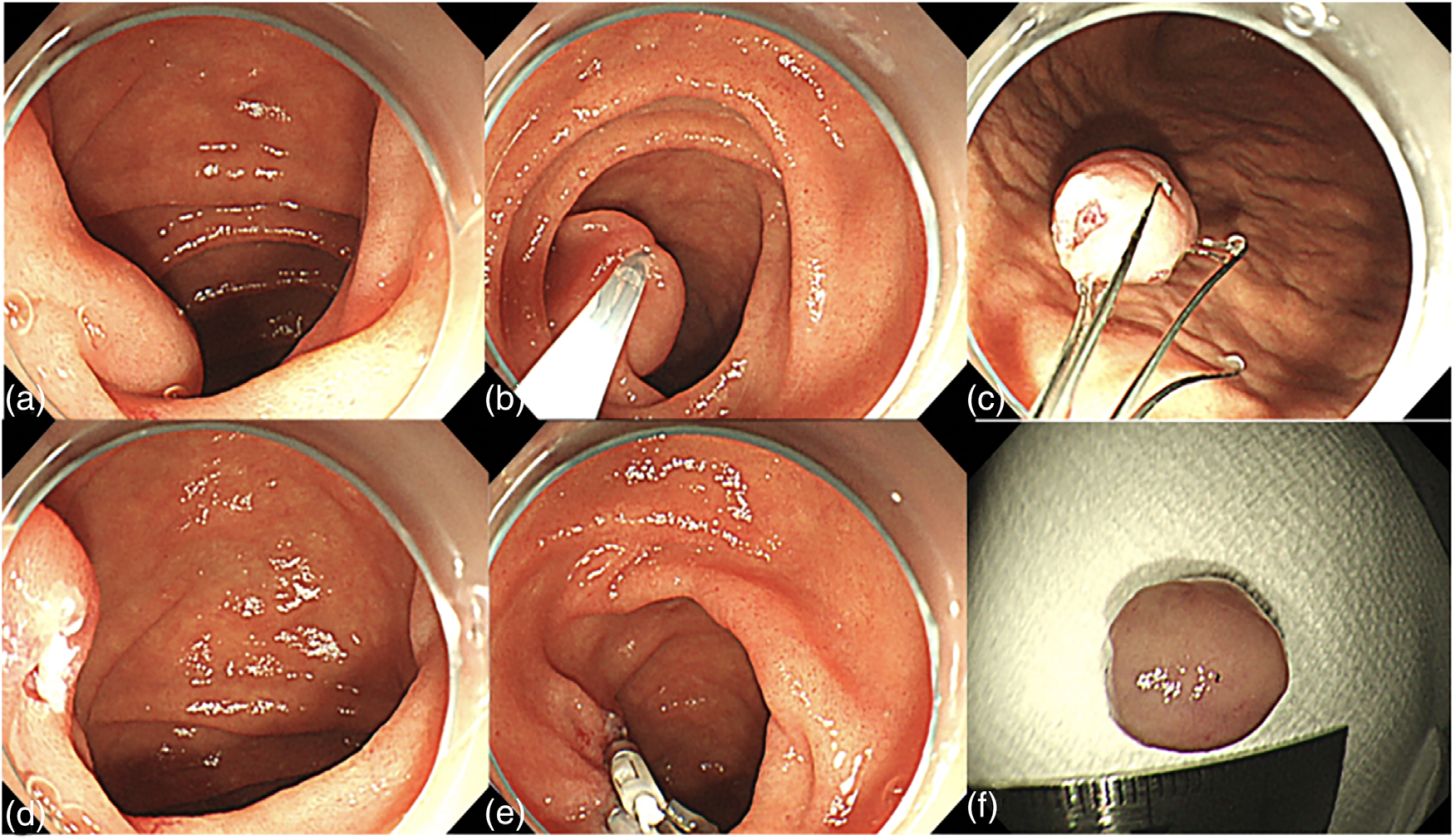
Endoscopic hot snare polypectomy procedural steps. (a) The stem of submucosal tumor was confirmed. (b) The tumor stem was ligated with a snare and energized by pure cut (VIO 3; Erbe) for excision. (c) The tumor was collected by foreign body removal forceps. (d) The cut end was endoscopically clear. (e) The cut end was closed with 5 clips and hemostasis was confirmed. (f) The excised tumor

Endoscopic ultrasonography (EUS) revealed a 10‐mm tumor with low echogenicity that included nodules and debris. The first layer of the cyst lumen was hyperechoic, the second layer was hypoechoic, the third layer was hyperechoic, the fourth layer was hypoechoic, and the fifth layer was hyperechoic, appearing to continue from the duodenal mucosa. The tumor did not communicate with the common bile duct or the pancreatic duct (Figure 2). An enhanced computed tomography (CT) scan of the abdomen revealed a cystic lesion with a diameter of 8 mm inside the duodenum at the level of the pancreatic head. Based on these examinations, a simple cyst, lipoma, lymphangioma, inverted intraluminal diverticulum, duodenal hamartomatous protrusion, and DDC were considered as differential diagnoses. However, the only diagnosis that should not be considered for resection was an inverted intraluminal diverticulum. To exclude this diagnosis, we confirmed that the cyst was cushion‐sign positive, squeeze‐sign negative. Furthermore, an enhanced CT scan of the abdomen taken in advance confirmed that the cyst was located inside the duodenum and that the duodenum wall was complete. After confirming the safety of resection, hot snare polypectomy was performed for diagnosis and treatment. A GI video scope (Olympus GIF‐Q260J; Olympus, Tokyo, Japan) was used in all procedures with no injections. The snare (SnareMaster 25mm; Olympus) was closed to capture the stem of the lesion. The lesion was then resected with a standard snare excision technique. The lesion was completely removed. The cut end was closed by five clips (EZ clip; Olympus) and hemostasis was confirmed (Figure 3). The post‐treatment course was uneventful, and the patient was discharged 6 days after treatment.

**FIGURE 4:**
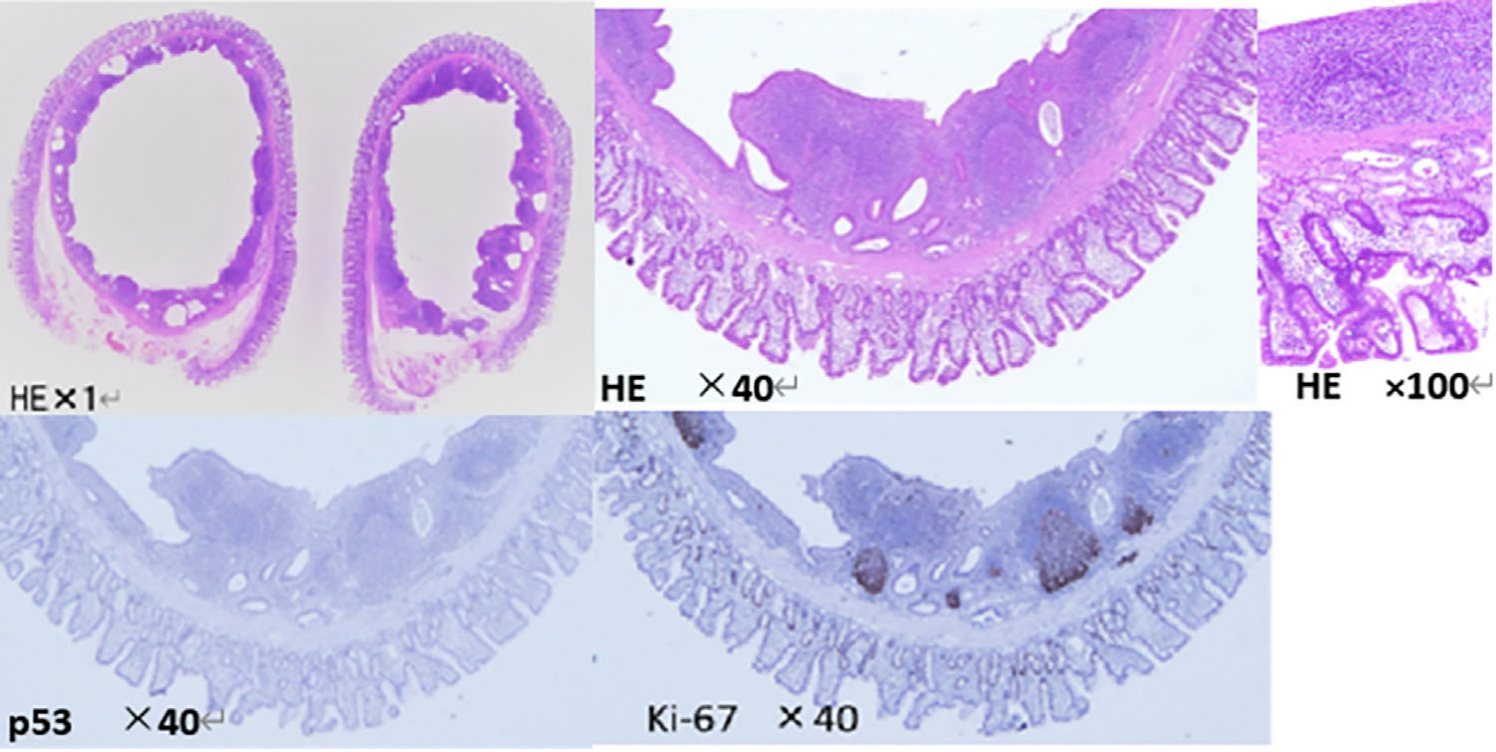
Histologic examination of the duodenal duplication cyst. The lesion had a cystic structure. The cystic space was lined by columnar epithelial mucosa and had a muscle layer. The inner and outer surfaces were formed by the normal duodenal mucosa. The solid component indicated by endoscopic ultrasonography was the inner layer of the cyst. HE: hematoxylin and eosin

Histologically, the lesion had a cystic structure. The cystic space was lined by columnar epithelial mucosa and had a muscle layer. The inner and outer surfaces of the cyst were formed by normal duodenal mucosa. Moreover, the cystic wall was highly infiltrated by lymphocytes, which led to the pathological diagnosis of DDC. The tumor was p53(−) and Ki76(+) by immunohistochemistry, indicating the presence of atypical regenerative epithelium in the inner layer. There was no sign of malignancy (Figure 4).

## DISCUSSION

A duplication cyst is a rare congenital anomaly formed during embryonic development of the alimentary tract. Duplication cysts are characterized by spherical or tubular structures which possess a well‐developed smooth muscle layer and are lined with a mucous membrane.^4^ The majority of DDC cases are diagnosed in infancy or early childhood. In rare cases, DDC can remain asymptomatic until adulthood, and 38% of patients with DDC are ≥20 years old.^1^ DDCs are predominantly located in the descending and inferior portion of the duodenum and sometimes communicate with the pancreatic or biliary duct.^1^ The treatment strategy is determined by the size and location of the lesion and whether it communicates with the biliary tree. Ectopic mucosa is found in 25%–35% of duplication cysts,^5^ and the presence of ectopic gastric mucosa can cause complications, such as bleeding and peptic ulcers. Furthermore, the presence of ectopic gastric tissue may cause malignant degeneration.^1^

The diagnosis of malignant GIDCs is rare; only a small number of cases have been reported.^6^, ^7^, ^8^ GIDCs occur more frequently in the colon, and the reported malignancy is up to 68%, whereas the malignancy rate of ileal cysts is 23%.^7^ In a recent meta‐analysis of 47 cases of DDCs, three cases developed malignancy.^3^ Additionally, Hata et al. reported a case of asymptomatic DDC with a carcinoid tumor.^9^ Therefore, even asymptomatic DDC may need to be treated to prevent malignant transformation and deny any possibility of neoplastic development.

Complete surgical resection is considered the optimal treatment for DDCs; however, pancreaticoduodenectomy is sometimes necessary. This procedure has high complication and mortality rates and should be carefully considered. A recent study suggested that DDCs could be safely resected endoscopically.^10^

In our case, the patient was an adult and asymptomatic. EUS was used to perform a close visual inspection; although the findings were insufficient for diagnosis, the differential diagnosis included a simple cyst, lipoma, lymphangioma, and duodenal hamartomatous protrusion. The tumor included nodules; thus, the possibility of malignancy could not be ruled out. Because the EUS image confirmed that the tumor had a long stem and was situated sufficiently far from the papilla of Vater, hot snare polypectomy was deemed appropriate. Furthermore, we had not definitively diagnosed the tumor as a DDC; thus, marsupialization was not an appropriate choice at that time. Furthermore, endoscopic resection allowed for detailed pathologic evaluation, which is an important advantage over marsupialization. Therefore, we decided that the DDC could be easily and safely treated by endoscopic resection, and a hot snare polypectomy was performed. The outcome was favorable, and no complications, including perforation, occurred.

## CONCLUSIONS

Endoscopic resection is a safe, effective, and minimally invasive alternative to surgical resection for small DDCs with malignant potential.

## CONFLICT OF INTEREST

The authors declare no conflict of interest.

## FUNDING INFORMATION

None.

## ETHICS STATEMENT

Not applicable.

## CONSENT FOR PUBLICATION

Written informed consent was obtained from the patient for publication of this case report and any accompanying images.

## Data Availability

All data generated or analyzed during this study are included in this published article.
